# A NOVEL APPLICATION OF BOLCK-CROON-HAGENAARS METHOD WITH MIXED-EFFECT LATENT CLASS ANALYSIS OF AGING TRAJECTORIES

**DOI:** 10.1093/geroni/igae098.2183

**Published:** 2024-12-31

**Authors:** Weiyi Xia, Anum Zafar, Olga Jarrín, Soko Setoguchi, Jason Roy, Haiqun Lin

**Affiliations:** Rutgers, The State University of New Jersey, Piscataway, New Jersey, United States; Rutgers, The State University of New Jersey, New Brunswick, New Jersey, United States; Rutgers, The State University of New Jersey, New Brunswick, New Jersey, United States; Rutgers, The State University of New Jersey, New Brunswick, New Jersey, United States; Rutgers, The State University of New Jersey, Piscataway, New Jersey, United States; Rutgers, The State University of New Jersey, Newark, New Jersey, United States

## Abstract

This work assesses the association between longitudinal trajectory classes and risk factors. The longitudinal trajectory classes are derived from the latent class analysis of longitudinal variables of interest such as repeated cognitive status and days in a quarter in a care setting during last years of life. This work aims to apply the Bolck–Croon–Hagenaars (BCH) method to the mixed effect group-based trajectory model to account for the individual heterogeneous effects. First, we fit a group-based trajectory model with random intercepts. The inclusion of the random effects poses a computational challenge that we address in this work. Next, each individual would be assigned a class membership with the highest posterior probability (modal assignment). Proportional assignments can be used as well. BCH weights are obtained based on the product of assigned class membership probabilities and the inverse of the matrix for misclassification errors. Finally, we reweight the data to assess the relationship between latent class memberships and risk factors. We test the performance of the bias-corrected BCH approach with simulated data. The simulation results reveal much smaller bias with BCH and larger bias with the naive three-step approach. We then apply the BCH method to assess the association between the aging trajectories, such as cognitive function trajectories or care setting trajectories in last years of life, and socio-demographic and clinical factors among Medicare beneficiary decedents. The application of the BCH method on the mixed-effect group-based trajectory modeling provides a rigorous assessment of the association between aging trajectories and risk factors.

